# African Immigrant Women's Maternal Health Experiences in Clarkston, Georgia: A Qualitative Study

**DOI:** 10.1089/whr.2023.0062

**Published:** 2023-12-12

**Authors:** Ehiremen Adesua Azugbene, Llewellyn J. Cornelius, Crista E. Johnson-Agbakwu

**Affiliations:** ^1^ Maternal and Child Health Translational Research Team (MCHTRT), College of Public Health Solutions, Arizona State University, Phoenix, Arizona, USA.; ^2^ School of Social Work, University of Georgia, Athens, Georgia, USA.; ^3^ Division of Preventive and Behavioral Medicine, UMass Chan Medical School, UMass Memorial Health, Worcester, Massachusetts, USA.

**Keywords:** maternal health, women's health African immigrants, migrant women, health knowledge, health experiences, health utilization, health services, female genital mutilation/cutting

## Abstract

**Introduction:**

The maternal health experiences of African immigrant women, their utilization of health care services, and the effects on maternal health have received limited attention in research. This research explored the maternal health experiences of African immigrant women residing in Clarkston, Georgia, and their use of health services.

**Methods:**

Fourteen African immigrant women responded to semistructured interviews. An adapted version of the Andersen health care utilization model explained the predisposing factors, enabling factors, and need factors, which influence the use of maternal health care for African immigrant women.

**Results:**

Findings were presented according to the Andersen health care utilization model. Analysis of the interviews resulted in 11 themes. The themes were as follows: (1) Community social structure, (2) community health beliefs, (3) health organization concerning the use of women, infants, and children, (4) social support at the individual level, (5) limited English proficiency, (6) need for better health education, (7) perception of care, (8) health financing, (9) long wait times and lack of transportation, (10) fear of medication and obstetrical interventions, and (11) impact of Female Genital Mutilation/Cutting.

**Discussion:**

Maternal health practices of African immigrant women are impacted by environmental and cultural factors. Public health interventions should be implemented to advance African immigrant women's health care utilization practices through required health education and tailored care, which will translate to positive maternal health experiences.

## Introduction

Maternal health care challenges are frequently encountered by women migrating from low- or middle-income countries in Africa to the United States.^1–6^ Immigrant women encounter various obstacles, including economic, social, linguistic, and cultural adjustments, when seeking access to suitable health care services.^6–9^ As a result of these barriers, African immigrant women report poor maternal health care utilization.^5,10,11^ They are less likely to receive preventive health screenings and access to care for health problems.^5,10,12^ African immigrant women have an elevated risk of postpartum depression, maternal death, preterm birth, and other adverse birth outcomes.^1,6,13,14^ The heightened risk may be associated with untreated conditions present before they migrated to the United States or cultural practices like Female Genital Mutilation/Cutting (FGM/C).^6,7,15,16^

Certain studies indicate a disproportionate rate of perinatal mortalities among African immigrant women, at a rate of 29.6/1,000, a phenomenon not solely accounted for by maternal risk factors.^4,15^ African immigrant women also have a higher risk of poor pregnancy outcomes due to lower rates of obstetric interventions.^1,14,17,18^ Therefore, the unique care needs of this population of immigrant women need to be explored to design interventions to improve their health outcomes.

African immigrants represent one of the most rapidly expanding immigrant groups in the United States, hailing from a diverse array of 51 countries with varying ethnicities, languages, and educational backgrounds.^19,20^ Over the past five decades, the influx of immigrants from African nations to the United States has witnessed a significant surge.^19–21^ To provide a perspective, the population of African-born Black immigrants residing in the United States stood at ∼560,000 in the year 2000. This figure experienced substantial growth, exceeding 1.9 million by the year 2019. This period spanning from 2000 to 2019 witnessed a threefold increase in the number of African immigrants making their way to the United States.^20^ This heightened migration trend can be attributed to a complex interplay of factors, including economic opportunities, sociopolitical dynamics, and religious motivations, as well as forced displacement due to conflicts in various regions across Africa.^19–22^

African nations account for ∼13% of the overall population of resettled refugees in the United States.^22^ Women make up 48% of the global refugee population.^23^ Refugee women, as a vulnerable subgroup of displaced individuals, have experienced involuntary displacement, have survived human rights violations, and require dedicated attention and support.^11,23^ Forced migration frequently leads to adverse impacts on the reproductive health of refugees. This problem often manifests in delays in accessing essential health care services and disparities in reproductive health outcomes.^11,23,24^ The increasing and multifaceted community of African women who have relocated to the United States necessitate the development of a research framework aimed at examining their health care experiences. This framework aims to provide insights that can enhance the provision of high-quality maternity care services.

This study explored the maternal health experiences and use of health services of African immigrant women living in Clarkston, Georgia. Clarkston, Georgia, serves as a resettlement community for immigrants of African origin, with most African immigrant residents in Clarkston originating from Ethiopia, Eritrea, Sudan, Somalia, and the Congo.^25^

**FIG. 1.**
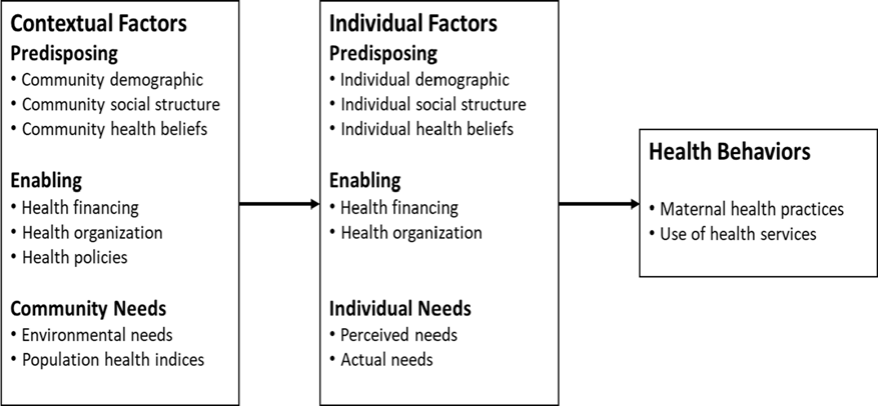
Andersen health care utilization model adaptation. Note: Adapted from Andersen health care utilization model.^58,59^

This study is grounded in a modified version of the Andersen health care utilization model,^26,27^ which elucidates the factors influencing health care utilization among African immigrant women, categorized under predisposing, enabling, and need factors, as well as health behaviors^26^ (Fig. 1). Originally developed by Andersen,^27^ this model explores how demographic, social, and economic characteristics impact families' use of health services. It underscores the significance of contextual factors, encompassing community attributes, health care environment, and provider-related elements, alongside individual characteristics that influence health care utilization.

These characteristics collectively encompass predisposing factors that incline individuals toward health care utilization, enabling factors that facilitate access to health care services, and the actual need for care.^26,28,29^ For a detailed delineation of these factors at both contextual and individual levels, please refer to Table 1,^6^ which defines and gives examples of predisposing, enabling, and need factors. The model also considers health behaviors, such as adherence to medical routines, self-care practices, and lifestyle choices, which are integral to the use of health care services.^26–29^ Specific examples of health behaviors within the scope of this study include the initiation and adherence to prenatal care services and other maternal health practices among African immigrant women. Furthermore, the model underscores how medical care, including ambulatory care and hospital inpatient services, predicts health care service utilization.^6,26^

**Table 1.** Adaptation from the Andersen Health Care Utilization Model: Definitions of Constructs and Examples

Construct	Definition	Examples of content
Contextual or community characteristics		
Predisposing characteristics	Factors from the community that inclines people to use or not use services.	**Community demographic**: A community with many young mothers, a community with many African immigrants. **Community social structure:** Communities where people live, and work that may be supportive or detrimental to their health and access to health services. Relevant measures include educational level, ethnic and racial composition, measures of spatial segregation, employment level, and crime rate, strong religious influence, NGOs that provide help to pregnant women. **Community health beliefs**: Community or organizational values and cultural norms and prevailing political perspectives regarding how health services should be organized, financed, and made accessible to the population. Beliefs about the roles of physicians, midwives, and other health care providers.
Enabling characteristics	Factors from the community that facilitate or impede the use of services.	**Health policy:** Recommendations, plans, and actions that are managed at the community level (public policies made in the legislative, executive, or judicial branch of government, at all levels from local to national) to achieve maternal health care goals in this population. **Health financing:** Community measures that suggest resources potentially available to pay for health services, including per capita community income and wealth **Health organization:** Amount and distribution of health services facilities and personnel to offer maternal services to African immigrant women. Examples are the ratios of physicians and hospital beds to population, office hours and location of service, provider mix, utilization, quality control, oversight, and outreach and education programs.
Need characteristics	Factors from the community that laypeople and/or professionals recognize as requiring medical treatment.	**Environmental needs:** Refers to health-related measures of the physical environment, among them the quality of housing, water, and air **Population health indices:** The number of pregnant African immigrant women in the community, or the prevalence of existing or pre-existing conditions that affect the maternal health of African immigrant women.
Individual characteristics		
Predisposing characteristics	Factors from the individual that incline the person to use or not use services.	**Individual demographic**: The ethnicity, age, education level, income level, marital status, the occupation of the African immigrant woman **Individual social structure**: Family and other personal support or social networks that provide help for pregnant women. **Individual health beliefs**: Attitudes, values, and knowledge that people have about health and health services that might influence perceptions of the need and use of health services such as health knowledge, religious beliefs, health literacy, and personal beliefs about maternal health care.
Enabling characteristics	Factors from the individual that facilitate or impede the use of services.	**Health financing:** Personal income, wealth, or health insurance that facilitates access to health care. **Health Organization** of health services for the individual describes whether or not the individual has a regular source of care or medical home and the nature of that source (private doctor, community clinic, or emergency room). It also includes means of transportation, reported travel time, and waiting time for care.
Need characteristics	Conditions that laypeople (perceived needs) and/or professionals (evaluated needs) recognize requiring medical treatment.	**Perceived need** can refer to how people experience and emotionally respond to symptoms of illness, pain, and worry about their health condition **Evaluated need** represents professional judgment and objective measurement of a patient's physical status and the need for medical care (blood pressure readings, temperature, blood cell count, *etc.*) These factors can refer to the status of pregnancy, the presence of female genital mutilation, and existing or preexisting conditions of ill health.

NGOs, nongovernmental organizations.

## Methods

This study was conducted with the assistance of a community-based organization in Clarkston, Georgia, working with African immigrant women. The Clarkston community in Georgia is a resettlement area for recent immigrants, especially immigrants from African countries. A convenience sample of 14 recent African immigrant mothers who have experiences with the use of health services in the United States participated in this study. The data were collected from September 2018 to February 2019. The inclusion criteria were as follows: (1) immigrant women from Africa, (2) between 18–45 years of age, and (3) who migrated to the United States within the past 6 years.

African immigrant women who participated in the study responded to semistructured interviews (Interview Guide Appendix). The interview guide was structured to address three overarching research questions. The research questions were as follows:

(1) What are the maternal health experiences of African Immigrant women regarding health care utilization?
(2) What contextual and individual factors facilitate or hinder maternal health care utilization?
(3) How do their maternal health experiences influence health care utilization?

The interviews were conducted by the lead investigator of the study at the office of the community-based organization assisting with the invitation of study participants. This location was safe, suitable, and confidential for the women and the research inquiry. Following the signing of the informed consent, the interviews were administered to the participants. Language interpreters in Arabic, Swahili, and Tigrinya assisted in the conduct of interviews when necessary. Other participants were interviewed in the English language. The interviews were audio-recorded, transcribed, and analyzed by the study investigator. The names of the participants were kept confidential as the women were assigned pseudonyms for analytical purposes. The University of Georgia's Institutional Review Board approved all aspects of this study (Protocol ID# MOD00006416).

Thematic analysis was used to analyze the interviews. Through its theoretical freedom, thematic analysis provides a flexible and useful research tool, which can potentially provide a rich and detailed, yet complex, account of data.^30^ Themes from the statements based on the interviews relevant to the lived experience of inquiry were developed into descriptions of the experience.^31,32^ The study investigator organized and coded the interviews with the aid of the NVIVO software program. The interviews were uploaded into the program after they were transcribed. The data were reduced to create codes that generated structured categories and subsequently, concepts or themes following thematic analysis.^33^ Themes from the coded statements relevant to the lived experience of inquiry were developed into descriptions of the experience.^31^

## Results

Fourteen African immigrant women participated in the study. Participants in all the interviews responded to questions to the best of their abilities, but needed frequent probing questions. Six of the 14 women who participated in the study were originally from Sudan, three were from Congo, one from Ethiopia, two from Eritrea, one was from Egypt, and one from Nigeria. In terms of educational attainment, six of the participants had less than a high school education and seven participants had some high school education, and were residents in the United States on average for 4.5 years (Table 2).

**Table 2.** Demographic Characteristics (*n* = 14)

Pseudonym	Country	Age	Income/year	Education	Language	#Years in the US
P (1)	Congo	32	<$20,000	Elementary	Swahili	2
P (2)	Sudan	36	$20,000–$34,999	Some HS	English/Arabic	4
P (3)	Sudan	33	<$20,000	Elementary	Arabic	5
P (4)	Togo	44	<$20,000	Elementary	Ewe	6
P (5)	Sudan	38	$20,000–$34,999	Some HS	English/Arabic	6
P (6)	Sudan	43	<$20,000	Elementary	Arabic	6
P (7)	Sudan	37	<$20,000	HS diploma	English/Arabic	5
P (8)	Egypt	38	<$20,000	Some HS	Arabic	3
P (9)	Congo	35	$20,000–$34,999	HS diploma	English/Swahili	4
P (10)	Congo	33	<$20,000	Elementary	Swahili	3
P (11)	Nigeria	34	$35,000–$49,999	Some College	English/Yoruba	6
P (12)	Sudan	23	$20,000–$34,999	Some HS	English/Arabic	2
P (13)	Eritrea	36	<$20,000	Elementary	Tigrinya	6
P (14)	Eritrea	28	$20,000–$34,999	Some HS	English/Tigrinya	5

Mean age = 35.2, mean number (#) years in the United States = 4.5.

HS, high school.

## Themes from the Interview

The themes resulting from the interviews are organized according to the contextual and individual factors of the Andersen health care utilization model that influenced the use of health services (see Table 3 for the model organization of themes). The analysis of interviews resulted in 11 themes. The themes are (1) community social structure, (2) community health beliefs, (3) health organization concerning the use of women, infants, and children (WIC), (4) social support at the individual level, (5) limited English proficiency, (6) need for better health education, (7) perception of care, (8) health financing, (9) long wait times and lack of transportation, (10) fear of medication and obstetrical interventions, and (11) impact of FGM/C.

**Table 3.** Themes from the Interviews

Construct of model	Themes
Contextual or community characteristics	
*Predisposing*: Factors from the community that inclines people to use or not use services.	*Community social structur*e of Clarkston with many immigrants, NGOs, and churches that help the community. *Community health beliefs* preference for female physicians and lack of support for preventive care
*Enabling*: Factors from the community that facilitates or impedes the use of services.	*Health Organization-WIC* was valued for maternal and childcare
Individual characteristics	
*Predisposing*: Factors from the individual that incline the person to use or not use services.	*Social support* provided by family and friends. *Limited English proficiency* that reduced understanding of medical personnel and required interpreters. *Need for Better Health Education* from health care providers about the progress of pregnancy and breastfeeding. *Perception of care with* regard to dismissive treatment from health care providers.
*Enabling*: Factors from the individual that facilitate or impede the use of services.	*Health financing* such as Medicaid and work health insurance facilitated access to health care. *Long wait times and lack of transportation* that limited access to care
*Need*: Conditions that laypeople (perceived needs) and/or professionals (evaluated needs) identify as requiring medical treatment.	*Fear of medication and obstetrical interventions* in terms of opposition to the use of medications such as epidurals during pregnancy and birthing processes. *Impact of female genital mutilation/cutting* and the effect on pregnancy and the ability of health providers to deal with the effects during pregnancy

WIC, women, infants, and children.

### Community social structure

The social structure of the community such as community-based organizations (CBO), nongovernmental organizations (NGOs), and churches, in Clarkston, Georgia, had a positive effect on the maternal health experiences of African immigrant women who live there. Clarkston is a city in DeKalb County, Georgia, with a population of 12,594, a median age of 28 years, and a median household income of $33,486, with a poverty rate of 36%. The population of Clarkston, GA, is 58% Black, 24% Asian, and 8% White.^25^ In 2016, the most common birthplace for the foreign-born residents of DeKalb County was Ethiopia, followed by Mexico and Myanmar.^25^

The countries of origin for most African immigrant residents in Clarkston are Ethiopia, Eritrea, Sudan, Somalia, and the Congo.^25^ Approximately 13% of the population of Clarkston (1,657 speakers) speak their native African language.^25^ A basic Google search indicates that there are about 20 churches or places of worship serving the Clarkston community. Also, about 30 nonprofits and NGOs work with the immigrant and refugee populations resident in Clarkston, Georgia, and Dekalb County.^34^

The assistance received from organizations in the community benefited the maternal health care utilization experiences of women in the study, as shown in the following interview excerpt: “They help the pregnant woman and baby. If you have a baby or you're pregnant, they help.”

Living in an area with large numbers of immigrants gave them access to support from members of their community, as shown in another interview excerpt:

“Clarkson is a very good place; I like Clarkson because it has different people from different countries. And you can find healthcare. When we came here, we didn't know anybody there. Only my husband went to the mosque here, and he found one Sudanese person there, he showed us Sudanese people's houses, and he took us to shop, and he helped us a lot. This means a lot to me.”

Other women received support from CBOs, NGOs, and churches that provide help to pregnant immigrant women. One woman highlighted the help she received from a CBO when she was pregnant and the assistance she received from a literacy program serving immigrants in Clarkston, Georgia:

“They help the pregnant women and babies. If you have a baby or you are pregnant, they will help you. A community health worker came to me and said she wanted to check on all the pregnant women and greet them. How are you doing? If you need my help, I will take you to the market.”

### Community health beliefs

Some women expressed beliefs about the roles of physicians, midwives, and other health care providers. They expressed their preference for female doctors and barriers to preventive care based on the values that are practiced in their home country. Two women talked about their preference for female doctors because of their religious and cultural beliefs, as shown in their interview excerpts. One stated, “I prefer a female doctor, it is difficult when the doctor is not a woman, he comes to check you inside, it feels shameful for my religion.” Similarly, the other indicated, “I like a woman doctor more than a man doctor because women do not show themselves to men in my culture.”

Several women disregarded the importance of preventive care and only visited the health center when they had a health problem. One of them stated,

“Sometimes I go to the hospital but not all the time. Sometimes once a year, sometimes after a year and three months, but only if I am sick or have to take care of my baby.”

The women believed that visiting the hospital was only important during ill health. Pregnancy was not considered ill-health in her culture and the use of prenatal or post-natal care would only be important if the woman or baby was ill.

### Health organization—WIC

The women did not give much information concerning contextual enabling characteristics that facilitated or impeded their use of health services. However, they discussed the impact of government programs such as WIC. This special supplemental nutrition program for WIC assists with food, health care referrals, and nutrition education to low-income pregnant, breastfeeding, and nonbreastfeeding postpartum women, and to infants and children.^35^ The overall impression women regarding the WIC program was that of appreciation. They were glad to have access to the program for themselves and their babies during their time of need. An example is shown in the interview excerpt:

“Yes, I'm still on WIC. After every three months, I go there and they give for the baby [and for me] to buy vegetables, milk, good stuff to buy for the baby. When you're pregnant you have for yourself and the baby, they give you two. After one year, they cut for you because they say after one year baby eats and after this, only for baby.”

### Social support at the individual level

Individual social structures—such as family members, friends, or social networks—facilitated the use of health services for all women who participated in the interviews. The women talked about support from friends, family members, and their spouses that facilitated access to transportation and food, which made it easier for them to manage their pregnancies. Friends from the country of origin were an important resource, as one of the women explained,

“My friends from the Sudanese community helped me when I was pregnant. My husband and two other family members helped me even after the baby had come. All my friends helped me a lot. At the hospital, the nurse helped me too.”

A second lady also discussed the help she received from friends during her pregnancy when she said:

The doctor told me that the baby will come on the 28th. So, I prepared myself… I call my friend who is a White lady. So, the lady took me to Farmers Market, a retail Farmers Market. We started to buy all the things that would need for pregnancy and baby.

### Limited English proficiency

Limited English proficiency was a barrier to the use of health services for most participants. This problem led to communication problems between the women and their health providers, particularly due to the limited understanding of medical language, which led to the need for interpreters. Seven participants in the interviews talked about the impact of their low English proficiency on their health care utilization. One lady discussed her communication problems with health care providers when she said,

“Yes, I understand because they know already, I'm a refugee, and my English is low, they know. If they tell me one thing and I don't understand, they should try to explain to me.” Big words. And I didn't understand the meaning. Because I only understand small words in English. And I said, “What is that? They make me worried a lot because I don't know the meaning.”

Four women also stated that access to interpreters at the hospital was helpful in their interactions with health care providers during their pregnancy and the lack of interpreters was a barrier to their use of health services. An example is shown in the excerpts from the interview with a woman who stated, “I don't understand the nurse, so they bring an interpreter.”

### Need for better health education

The women discussed barriers to accessing health information from health providers at the health care centers during their pregnancy. The women spoke about difficulties with getting access to information from the doctors as barriers to the use of health services. One woman stated,

“It is not about the language because they bring interpreters too, for me. If something is important, they would tell me, they would tell the interpreter too, but sometimes they didn't tell me.”

Some women talked about the need for information on pregnancy prevention and contraceptives because their last pregnancies were not planned, and this made health care during and after their pregnancies very difficult for them. One of them stated,

“I did not plan to have my last baby, but I did not know how to prevent pregnancy. When we came to the hospital, they said that there is a way to plan to have the baby when we want but I do not know how to get that information. Some of my friends need more help because a baby is expensive, but our people do not talk about it.”

The women also required information about how to handle problems with breastfeeding after pregnancy. Some of them had problems with breastfeeding and access to health information about how to care for themselves when they were breastfeeding their babies, as shown in the excerpts of one of the interviews,

“I wish I had more help with the bread feeding of the baby because it was difficult to make milk for the baby and everything was very painful but it was difficult for me and the baby …very difficult …it got better but at first, I could not get too much help with the problem.”

### Perception of care

Two participants discussed what they perceived as different treatment from health care providers. One of them expressed her discomfort with the perceived dismissive behavior from the doctors and nurses at the hospital when she went to receive care during her last pregnancy. She felt the health care providers did not attend to her properly because she could not speak English properly and did not have access to an interpreter. Excerpts from the interview:

“When I came new and my child, he was sick, and I don't know how to say anything, and they told me you must stay in here sitting down until you find somebody to talk to you…to translate to you… They behave this way because they don't understand me, and I don't understand them. I called my agency, and they have one man who speaks Arabic and he translated for me…They don't have an interpreter in the clinic.”

The other woman explained that the doctors and the nurses were indifferent to her during her visit to the hospital. She stated,

One time it happened. I said, “Excuse me,” I want to talk to them. And it's like a doctor and nurse together. They came to see me, and they just looked at me like this. Nobody touched me, nobody asked me any questions. They just turned around and they're talking together. And I said, “Excuse me, excuse me.” Nobody wants to hear me. They hear what I'm saying, but they don't want to talk to me. They wanna finish their talking first. After that, he turned to me and said, “Okay, sorry. What did you say?” And after that, I get mad. The nurse did that a lot of times… I said, better for you to go. Don't sit here, because you're not helping anybody.

Health knowledge about the progress of pregnancy was facilitated by access to ultrasounds and the detailed explanation of the meaning of the procedure to the participants. She stated in her interview,

“Oh. when I go here every month, you can see the baby, only with something like a headphone… After five months they sent me to another doctor… because this doctor's office doesn't have that.”

### Health financing

The women stated that access to health insurance was important in the way they used health services. Most of the women had access to health insurance in the form of Medicaid during pregnancy. Medicaid covers the mother and baby during the pregnancy, but does not cover the mother after pregnancy. Only the participant from Nigeria had access to private health insurance through the company insurance from her husband. Two others also had access to some health insurance from their husband's companies, in addition to support from Medicaid.

Other women talked about the difficulty with access to health insurance, which was a barrier to the use of health services, as shown in her interview excerpts:

“I started prenatal care when I was 6 months pregnant because I didn't have health insurance before. The organization helps me get Medicaid.”

Two of the women expressed their dissatisfaction with the way the hospital organized the billing for their health insurance. The women referred to inconsistencies with the way their bills were processed as financed under personal billing/health insurance or Medicaid, as shown in the excerpts from their interviews:

The insurance is important for me because I went to the hospital last time, and the doctor told me, “Your insurance is at the job you worked before the past three months… because we are not working there anymore, we cut it off. ‘So now go pay $80.’” I say, “Okay…The last time my nails, and then my legs were swollen, I went to the hospital, and the doctor said, ‘You'll pay $280.’ I paid $280, and they saw me. After I went back home, they sent me $364 again, and I paid. So, if you don't have insurance in America…it is killing people, I'm telling you… Also, they say I should pay $223 every month before they will help me pay the rest. If I work my money over that they will take it back from me. What is this? Are we going to survive it?.”

Another lady stated,

“They charged me with the Cigna, and I have like five bills. They came to me, and I don't have money. I went to the doctor and told him; this came from your office.”

### Long wait times and lack of transportation

Women spoke about practical barriers to receiving maternal health care. Some of the women spoke about the excessive hospital wait times and problems with transportation. One woman stated, “The bus comes late too much, sometimes 30 minutes late and I have to walk then” and I walk.”

Another participant also supported this claim when she stated,

“When you come new, they help us to go to the doctor and after that, we go by ourselves. You know after three months or, they say everything now you have to do everything by yourself, and I go with MARTA, you know the MARTA bus is not always on time.”

Another woman summarized what the others had said about the hospital wait time when she stated,

“I just go there to the hospital, and the first time I go there, the nurse asks me what happened. And I say my water broke. And just she told me, excuse me, go get a chair and wait… And they put the stuff on me, and they told me, give me your ID and give me your stuff. And I gave it to them. And after that, she told me, ‘You can go out. Somebody will meet you there and I am just there sitting and waiting.’”

### Fear of medication and obstetrical interventions

Some women referred to pregnancy as a normal process and opposed to the use of medications such as epidurals during labor and obstetrical interventions. They discussed the individual perceived need factors for care, such as response to pain and the use of obstetrical interventions during labor. They expressed dissatisfaction with the use of pain medication and fear of obstetric interventions during the birthing process. Some of the women had cesarean sections during their pregnancies and agreed to the procedure only after much personal contemplation. They strongly opposed to the use of pain medication or induced labor during pregnancy because it was not normal and had side effects that were detrimental to women after having their babies. Excerpts from interviews are shown below:

“I don't need it [the pain medication]. I know that if you have a baby, you will feel pain. When you have your baby… I don't like to get injections for my back to reduce the pain. After that, you have a problem tomorrow. I think so. I don't like it. One lady in my apartment complains every day…My back is paining me.” (Lady A)

“The pain [made it difficult to manage the pregnancy]. They asked me to take the medicine for the pain; I told them I didn't want that medicine because it was not good. The medicine they give you in the back, I tell them that is not good.

Some people tell me; my friends tell me that would affect your health. You will be like, you cannot walk. Sometimes you have pain in your feet and in your back always you have that pain if you get that medicine.” (Lady B)

One woman had low blood pressure during her pregnancy, which required her to have a cesarean section during her birthing process. She stated,

“I had to do the operation where they cut you open to have the baby but I was very afraid and ashamed. I was afraid because they say if they cut you open it is not good. Women in my culture are supposed to have babies the right way but not through the operation. But the doctor said this is the best way.”

### Impact of FGM/C

Three women who participated in the interviews discussed the impact of FGM/C and its effect on pregnancy. FGM/C was regarded as a condition that requires medical attention and a need factor for care during pregnancy. They discussed the inability of health providers to deal with the effects of FGM/C during pregnancy. One of them explained that, although they had undergone the process as a child, she did not experience any problem during pregnancy. Other women did not report a personal experience of FGM/C, but referred to friends who suffered from the procedure and had problems during their pregnancies. They stated that friends who had experienced the procedure were weary of dealing with doctors because the doctors did not know how to deal with the problem and often recommended cesarean sections during the birthing process, as shown in the interview excerpts:

“I did not have a problem with the FGM/C but some of my friends have done it from back home before they came here to live and have their babies. Some of them say the doctors treat them differently because of it. Some of them are afraid of the doctor saying they will cut them open because they have done it and are always not happy about the operation during the delivery of their baby…but me I did not do it so have no problem.”

## Discussion

This study is one of the few investigations conducted on the maternal health experiences of African immigrant women living in Clarkson, Georgia. The study is an important addition to the limited research on the maternal health of African immigrant women in the United States. The results of the interviews highlighted several factors that influence maternal health care for African immigrant women.

Social support was fundamental to pregnant women and new mothers for health care utilization. Social support refers to the provision of psychological and material resources from an individual's social networks to cope with stress. Social support can be instrumental (material support), informational (advice or guidance), and emotional (reassurance, care, or empathy).^36^ Social support provided by the community was valuable in the maternal health care that the women sought and received.

Living in Clarkston, Georgia, a community with large numbers of African immigrants, facilitated access to community-based organizations, NGOs, and other relevant support in the community for the maternal health of the women. Government programs such as WIC were a source of support women in the study and access to these programs should be encouraged for the people who need them. Social support at the individual level also affected the maternal health care utilization of participants in the study. Acculturation barriers for African immigrant women can be reduced through social and economic assistance programs.^4^

This study shows that resettlement communities for immigrants should be encouraged as this facilitates social support, access to health care, and the use of maternal health services, especially for recent immigrants. Individual, interpersonal, community, and organizational factors can adversely affect the health of immigrant women after resettlement.^37^ Immigrant refugee mothers struggle with the problem of living between two cultures, resulting from their unique experiences of forced migration and subsequent resettlement.^38^

These women often experience a climate of uncertainty as they navigate life transitions and challenges as they acclimate to the unfamiliar milieu of their new host country. They have to assimilate their beliefs with new societal values.^38^ The recommended maternal health practices of most Western nations are often different from the maternal practices of their host country.^38–40^ Immigrant women are faced with the challenge of re-evaluating or assimilating their beliefs with their host country.^38^ Future studies should focus on the interventions implemented to facilitate easier navigation and assimilations of maternal health care for African immigrant women.

Health beliefs about pregnancy (personal, cultural, and religious) influenced the maternal health utilization experiences of women in the study. Women who participated in the study expressed beliefs about the roles of physicians, midwives, and other health care providers. They preferred female doctors because of their cultural and religious beliefs. This narrative reinforces the perspective that African immigrant women strongly prefer female providers for gynecologic care in previous research.^4,18,41^ Thus, the assistance of female interpreters and providers at health centers will promote effective communication and access to health care services for African immigrant women.

Concerning the roles of health care providers, some women perceived the treatment from health care providers as poor. The women perceive that they receive dismissive or disrespectful treatment because they are immigrants and have difficulty expressing themselves to the doctors and nurses. These conclusions are aligned with previous research showing that African immigrants have the general perception that health providers discriminate against them because of their race and language and that they are less sensitive to their needs.^42,43^

This study shows that African immigrant women regard pregnancy as a normal process and are not open to obstetrical interventions during their pregnancies. Previous research regarding obstetrical interventions shows that African immigrant women felt that doctors were dismissive of their labor practices and recommended obstetrical intervention—like a cesarean section—too often, especially for women who have experienced FGM/C.^18,44,45^ The concerns of women regarding the inadequacy of health providers to deal with the problem of FGM/C resonates with the previous research on negative perceptions by health care providers.^7,16,46,47^ Health care providers were inadequately equipped to deal with the problem as they lacked knowledge, sensitivity, and empathy for women with FGM/C.^7,16,46,47^ Therefore, health care providers require adequate training and understanding of FGM/C^48,49^ to improve the maternal health experiences of African immigrant women.

African immigrant women's narratives of pain have not been adequately addressed by conventional medicine, as presented in Western societies.^4,41,50,51^ The maternal health experiences of African immigrant women will benefit positively from health education and information that address knowledge gaps in family planning and their narratives of pain. Lack of information about existing services and the cost of health services was also a barrier to care. African immigrant women require information to navigate the health system, especially regarding access to health insurance and transportation. This study reinforces the findings from previous research that access to health insurance, access to transportation, and low waiting times at health centers enabled African immigrant women to use health services.^4,40,43,52–54^

This study is also aligned with the conclusion of earlier studies in other Western developed countries, which supports the need for culturally competent maternal health care and new models of maternity care that address both individual and environmental factors for African immigrant women.^37,38,40,55–58^ African immigrant women face barriers when navigating complex systems that could affect their access to care, transportation, and health insurance.^37^ One study on the maternal health of African migrant women who emigrate to Western nations such as Spain shows that these women face institutional barriers when accessing appropriate health care.

Such barriers include the poor communication and discriminatory attitudes of health care providers and the need for culturally appropriate health systems.^56^ Another systematic review of the experience of maternity care by immigrant women in the United Kingdom showed that African immigrant women avoided utilizing maternity care because they perceived health professionals as discriminatory and indifferent to their cultural and social needs.

The research highlighted the need for effective and culturally sensitive health interventions for the maternal health of immigrant women.^58^ The results of a systematic review on the health needs of migrant women from FGM/C–practicing countries in high-income contexts recommend that maternal care integrate cultural safety, foster patient-provider trust, and engage women affected by FGM/C to design and provide quality care that is tailored to their needs.^57^ Health care providers require training on how to interact with women who have undergone this procedure without stigmatizing or eroding their dignity, but instead provide appropriate health care when the situation arises.^37^

One major recommendation from the results of the study is the salient need for the cultural tailoring of public health interventions to meet the needs of diverse communities. The American College of Obstetricians and Gynecologists (ACOG) advocates for access to maternal health care and tailored care for underserved and immigrant populations.^59^ ACOG advocates for the advancement of culturally sensitive obstetric and gynecologic care environments. This entails demonstrating respect for cultural health beliefs and traditions and providing interpreters and materials in languages that align with the patient population's needs.^59^

In this study, some women noted that they appreciated the help of interpreters during their health care experiences, while others complained about the lack of inadequacy of such services. This promotion of culturally inclusive obstetric and gynecologic care is also central to the National Standards for Culturally and Linguistically Appropriate Services (CLAS) standards in health care.^60^ CLAS reduce health disparities and achieve health equity through improved quality of services provided to all individuals. Respect and responsiveness to the maternal health needs of African immigrant women are central to improving their maternal health experiences and overall health outcomes. Ultimately, collaborations between health care organizations, health care providers, policymakers, and researchers are necessary to implement more culturally relevant maternity care policies and management interventions that advance the health of immigrant communities.^61^

Communities across the United States, like Clarkston, Georgia, are growing in language and cultural diversity.^25,62^ Therefore, communities like Clarkston can benefit from applying what has worked in large diverse communities to address their complex health needs. Queens County, New York, is an example of a community that has succeeded in responding to cultural diversity. Queens County, New York, is called the diversity capital of the world as more than 138 languages are spoken in the county.^63^

In response to this extensive diversity, health care providers and organizations should integrate ethnically diverse health care staff who are trained in cultural nuances and the language diversity of the targeted community, as well as staff who are up to date on immigration policies and procedures. Also, the use of community health worker models^64–66^ for advancing maternal health care is recommended for advancing culturally competent care for African immigrant women. In terms of access to health care, transportation was one issue that came up in the results of the study as a barrier to care for the African immigrant women in Clarkston. Georgia. A simple Google search and one study^62^ on the maternal health of immigrant populations of Clarkston show that the only major health provider close to the city is located at the Emory Decatur Hospital.

The health care system in the city of Queens, New York, has three satellite clinics, in addition to the hospital-based outpatient obstetrics and gynecology clinic that caters to the maternal health needs of the population.^67^ Other major hospital-based clinics also serve the population and women with equipped and culturally sensitive obstetrical and gynecological care for emergency, primary, and preventative inpatient and outpatient activities.^67^ Mirroring the maternal health interventions of cities like Queens, New York, would make it beneficial to set up equipped satellite health care centers closer to the populations that need them.^68^ The creation of satellite health centers that mirror the services of the larger health centers will serve to prevent problems like transportation that could affect access to care.

This study had some limitations. Challenges could occur during the interview process such as how to phrase and negotiate questions, concerns with the actions and biases of the interviewer, unanticipated behaviors from participants, and poor recollection of information..^32,69,70^ The participants could also have been careful in their responses to the interview questions due to the sensitive issues discussed in the interview. Some participants in the study were not too comfortable with discussing the problem of FGM/C on a personal level.

Also, the reduced English proficiency and the language barrier in some of the participants may have affected the quality and participant honesty in interviews because of the use of interpreters. Furthermore, research has demonstrated the influence of health literacy on the health care experiences and utilization patterns of African immigrant women,^1,6,53,71^ particularly within specific communities like Somali African immigrants.^53^ As a result, there is a pressing need for more extensive research on a broader scale, encompassing African immigrant women at large, to explore the ramifications of health literacy on their maternal health care utilization requirements.

## Conclusion

This study significantly contributes to the field of maternal and child health research, especially for African immigrant women living in Clarkston, Georgia. The findings of the study support the encouragement of resettlement communities for immigrants as they facilitate access to social support because living in Clarkston, Georgia, was beneficial to women in the study. Based on the results of the study, health providers require training on the cultural norms of African immigrant women to address barriers to care.

Also, public health interventions should be put in place to help African immigrant women have access to required education and tailored care that address the fear of pain medication, obstetrical interventions, FGM/C, and the need for family planning. These interventions will also help this population to navigate the health system, especially on complicated topics such as obstetrical interventions and health insurance. In addition to the Andersen model, more research is required regarding socioecological frameworks, exploring the sociopolitical determinants of health that impact maternity care for African immigrant women. Future studies on the maternal health experiences and health care utilization of African immigrant women should consider socioecological contexts that influence their maternal care experiences and health-seeking behaviors.

## Supplementary Material

Supplemental data

## About the Authors

**Ehiremen Adesua Azugbene, PhD, MPH, CHES,** is an academic and research expert in health promotion, health behavior, and disease prevention. She is a Presidential Postdoctoral Scholar at the College of Health Solutions working with the Maternal and Child Health Translational Research Team (MCHTRT) at Arizona State University. Her research is focused on maternal and child health, especially in underserved populations. Her research also covers health policy, health services, health literacy, global health, and immigrant health. She is competent in qualitative, quantitative, and mixed-methods research. **Llewellyn J. Cornelius, PhD,** is an accomplished researcher and a Donald L. Hollowell Distinguished Professor of Social Justice and Civil Rights Studies at the University of Georgia. He has worked in tandem with researchers, administrators, and consumers in the design, implementation, and evaluation of interventions focused on improving the health and well-being of under-resourced communities. He specializes in research methods in social work, survey research, ethnocultural qualitative research, meta-analysis, and social policy. His research work also covers racial/ethnic health disparities, research methods, social determinants of health, development of change interventions, and examination of barriers to the adoption of interventions. **Crista E. Johnson-Agbakwu, MD, MSc,** an Obstetrician/Gynecologist, is the Executive Director of the Collaborative in Health Equity at the University of Massachusetts Chan Medical School. Her research focuses on investigating strategies to advance sexual and reproductive health equity for women of color, including migrant women, to improve health care access and utilization, sexual and reproductive health education, counseling, and community engagement to enhance healthcare provider cultural competency. She has spearheaded a unique patient-centered medical home for migrant families at a public safety net teaching hospital and helped facilitate the care for over 16,000 migrant families hailing from 68 countries across sub-Saharan Africa, South-East Asia, and the Middle East and speaking over 71 languages. She has led a federally funded effort to improve health care services, community, engagement, and provider cultural competency on FGM/C, and gender-based violence, and has provided consultative expertise to the CDC and WHO on refugee women's health and FGM/C.

## Supplementary Material

Interview Guide Appendix

## Abbreviations Used

ACOG: American College of Obstetricians and Gynecologists
CLAS: Culturally and Linguistically Appropriate Services
CBO: community-based organizations
FGM/C: Female Genital Mutilation/Cutting
HS: high school
NGOs: nongovernmental organizations
WIC: women, infants, and children
