# Supplementary material for: African Immigrant Women's Maternal Health Experiences in Clarkston, Georgia: A Qualitative Study
Source: Womens Health Rep (New Rochelle). 2023 Dec 12;4(1):603–16. doi: 10.1089/whr.2023.0062 (PMC10754423; doi:10.1089/whr.2023.0062)
Supplement: Supplemental data [file Suppl_Interview-Guide-Appendix.docx]

**African Immigrant Women’s Maternal Health Experiences**

**Interview Guide**

1. Can you tell me about your experience with the birth of your last child?

Probe: Please talk more about your birth experiences in the last 6 years

1. Can you talk about any maternal services that you have received in the United States?
2. Tell me about the kind of care and information you received.

Probe: Can you tell me if the information was easy to understand and easy for you to use?

1. What kind of problems did you have with getting maternal health information and using the information?

Probe: What other information or services would you have liked?

1. Can you talk about a time you felt you were being treated differently in the process of seeking maternal or child health care services?

Probe: What do you think was the reason for the difference in treatment that you experienced?

1. How has living in the United States as a recent African immigrant affected your access to maternal health services in the United States?
2. How did having health insurance or not having health insurance affect your access to maternal health services?

Probe: How has being able to pay for hospital services affected your decision to go to the hospital?

1. How did your experience with access to care affect your overall experience and decision towards seeking maternal care?
2. In your decision to seek maternal care or use hospital services during your last pregnancy, what are some factors that made you seek care?

Probe: How early and how often did you seek care or use hospital services?

1. What kind of support have you received from family, friends, and other avenues before, during, and after having your baby?

Probe: Tell me about support with transportation, food, and any other help from family, friends, the community, or the government.

1. How has the support you received affected the way you use health services in the United States?

Probe: Has the support made it easier for you to access these services or there was not much difference?

1. What are your personal and cultural beliefs about pregnancy and maternal health?

Probe: How do these beliefs affect the way you use maternal health services in the United States?

1. Have any prior existing health conditions such as female genital cutting, or any other conditions affected the way you used hospital services during your last pregnancy or pregnancies in the United States?
2. Tell me more about your maternal health experiences after the birth of your child.

Probe: Talk more about your health and the health of your baby after the birth of your child. What services were helpful to you and the baby?

1. Is there anything else you want to share with me concerning your healthcare before, during, or after your pregnancy?
2. Do you have any questions for me relating to this study?
